# Prevalence and associated factors of cognitive frailty in older patients with chronic kidney disease: a cross-sectional study

**DOI:** 10.1186/s12877-022-03366-z

**Published:** 2022-08-17

**Authors:** Jing Chang, Wenwen Hou, Yanchun Li, Shujuan Li, Kai Zhao, Yanfei Wang, Yuanping Hou, Qianmei Sun

**Affiliations:** 1 Department of Internal Medicine, Beijing Chaoyang Hospital, Capital Medical University, Gong-Ti South Road 8#, Chao-Yang District, Beijing, 100020 China; 2 Department of Nephrology, Beijing Chaoyang Hospital, Capital Medical University, Beijing, China; 3 Department of Neurology, Beijing Chaoyang Hospital, Capital Medical University, Beijing, China

**Keywords:** Cognitive frailty, Chronic kidney disease, Elderly, Risk factors, Nomogram

## Abstract

**Background:**

Chronic kidney disease (CKD) is prevalent in older adults. In the aging CKD population, cognitive frailty is more common, but its prevalence and associated risk factors need to be further investigated.

**Methods:**

This is a cross-sectional study that enrolled patients aged ≥ 60 years with a diagnosis of CKD from January 2018 to February 2021. Patients were assessed for frailty and cognition with the FRAIL and the Mini-Mental State Examination (MMSE) scales and were divided into the cognitive frailty and non-cognitive frailty groups. Risk factors for cognitive frailty were identified by univariate and multivariate logistic regression analyses. A prediction model for cognitive frailty was built and a nomogram was plotted. The performance of the nomogram was evaluated by using a concordance index (C-index) and calibration plots.

**Results:**

A total of 1015 older patients with CKD were enrolled, among whom 607 (59.8%) were males and 408 (40.2%) were females, with an age ranging from 60 to 98 years, and an cognitive frailty prevalence of 15.2%. The prevalence of cognitive frailty varied among the CKD stages 1–5, with rates of 4.7%, 7.5%, 13.8%, 18.5%, and 21.4%, respectively. Multivariate logistic regression analysis showed that age (OR = 1.11, 95%CI 1.08–1.14, *p* < 0.001), depression (OR = 2.52, 95%CI 1.54–4.11, *p* < 0.001), low social support (OR = 2.08, 95%CI 1.28–3.39, *p* = 0.003), Charlson comorbidity index (CCI) (OR = 1.92, 95%CI 1.70–2.18, *p* < 0.001), eGFR (OR = 0.98, 95%CI 0.96–0.99, *p* < 0.001) and albuminuria (OR = 5.93, 95%CI 3.28–10.74, *p* < 0.001) were independent risk factors affecting the association with cognitive frailty in older patients with CKD. A nomogram for assessing cognitive frailty was established and well-calibrated with a C-index of 0.91 (95%CI 0.89–0.94).

**Conclusions:**

The prevalence of cognitive frailty was higher in older patients having CKD. Advanced age, comorbidity, depression, low social support, eGFR and albuminuria were independent risk factors for CKD accompanied with cognitive frailty.

## Background

With the progress of medical technology and increased life expectancy, population aging is becoming an evident reality. Recent data have shown that number of Chinese people aged 60 years and above reaches 264 million in 2021, which accounts for 18.70% of the total population. There are 190 million people aged more than 65 years, attributing to 13.5% of the overall population [1]. The health of older adults vary greatly, Many older adults are not in good health despite just entering their mature years, while some are very hale and hearty. Therefore, age cannot fully reflect the health status of older people. Frailty, in turn, indicates a decline in reserve capacity and anti-stress ability of an organism, standing as an important indicator of health status [2–4].

Older people usually experience comorbidities and endure functional decline, leading to frailty and cognitive impairments in a high proportion of the older population. Frailty and cognitive impairment interact in a vicious cycle, which can increase the risk of adverse outcomes, such as disability, falling, hospitalization, and death [5, 6]. In this context, the International Academy on Nutrition and Aging (IANA) and the International Association of Gerontology and Geriatrics (IAGG) jointly proposed the concept of cognitive frailty in 2013 [7]. Cognitive frailty is defined as frailty and cognitive dysfunction without a definitive clinical diagnosis of dementia. Cognitive frailty poses a serious threat to the quality of life and health of the elderly population. In recent years, researchers have intensified their studies in this field. A study by Ruan et al. [8] found that cognitive frailty was a pathological state, which was a precursor to brain aging and neurodegeneration. A meta-analysis including 14 studies with 57,559 older adults suggested that the prevalence of cognitive frailty was 2.5%-50% [9].

Likewise, the prevalence of chronic kidney disease (CKD) increases with age. Previous studies have shown that CKD affects about 119.5 million Chinese individuals, with an overall prevalence rate of 10.8% and a higher prevalence of 33% in people aged 60–89 years [10]. Because CKD patients suffer from chronic inflammation, cardiovascular diseases, metabolic disorders, malnutrition, anemia, and other conditions, a higher incidence of physical and cognitive frailty is observed in these patients than in the general population. If cognitive frailty can be screened out in the early stages of CKD, successful aging and independence in the clinical prognosis of CKD patients may be achieved [11]. Therefore, it is important to assess the prevalence of cognitive frailty in older adults with CKD and investigate the risk factors associated with this disease.

## Methods

### Study design and population

In this study, a cross-sectional survey was conducted. Subjects admitted as inpatients at the Department of Internal Medicine and the Department of Nephrology, Beijing Chaoyang Hospital, Capital Medical University, from January 2018 to February 2021, were enrolled. Patients aged 60 years or above who were diagnosed with CKD met the inclusion criteria. According to the Kidney Disease Improving Global Outcomes (KIDIGO) guideline, a patient was diagnosed with CKD when he/she had abnormal kidney structure or function for more than 3 months, which had an impact on health, and an estimated glomerular filtration rate (eGFR) below 60 mL/min/1.73 m^2^, or developed albuminuria [12]. The exclusion criteria were: 1) those with severe visual, hearing, or verbal communication impairments, or unable to answer the questionnaire; and 2) patients diagnosed with Alzheimer’s disease, dementia or other psychiatric illness. The experimental protocol was reviewed and unanimously approved by the Medical Ethics Committee of Beijing Chaoyang Hospital, Capital Medical University, Beijing, China (approval number 2017-ke-98). All patients signed an informed consent form. Patient who was illiteracy signed the informed consent from the legally authorized representative or from the guardians.

### Diagnosis and definitions

Patients with both frailty and cognitive impairment were considered to suffer cognitive frailty, while those without both complications were regarded as having no cognitive frailty. The patients were divided into the cognitive frailty group and the non-cognitive frailty group, according to whether they developed cognitive frailty. Frailty assessed by the FRAIL scale, which consisted of 5 aspects: fatigue, resistance, ambulation, illness, and weight loss. Fatigue was defined as whether the patient endured most or all of the frailty in the past month. Resistance referred to inquiring whether the subjects had difficulty going up one floor alone without resting. Ambulation referred to an assessment of how difficult it was to walk a block alone. Illness referred to the test of the presence of 5 abnormalities of the following conditions: hypertension, diabetes, malignancy, chronic lung disease, asthma, acute heart attack, congestive heart failure, angina, arthritis, stroke, and kidney disease. Weight loss examined whether the reduction in weight was greater than 3 kg or ≥ 5% in the past year. Each item was scored as 1 point, and the maximum overall score was 5. A score of ≥ 3 indicated frailty [13]. Cognitive assessment was performed using the Mini-Mental State Examination (MMSE) scale, with a score ≥ 30 suggesting a conserved cognitive function. Since MMSE scores were influenced by educational level, cognitive decline was defined as illiteracy MMSE ≤ 17, elementary school graduate MMSE ≤ 20, middle school graduate MMSE ≤ 22, and higher education graduate MMSE ≤ 24 [14].

### Clinical data collection

Patients’ demographic information (including height, weight, etc.) and general clinical data (e.g., blood and biochemical routines, past medical history, etc.) were collected. Body mass index (BMI) was calculated by dividing body weight (kg) by the square of height (m). CKD-EPI formula was applied to calculate eGFR [15]. CKD stages 1–5 were defined as eGFR ≥ 90 mL/min/1.73m2 with albuminuria, 60–89 mL/min/1.73m2 with albuminuria, 30–59 mL/min/1.73m^2^, 15–29 mL/min/1.73m^2^, and < 15 mL/min/1.73m^2^, respectively [12]. The circumference of the thickest part of the mid-calf was measured with a soft ruler and recorded as calf circumference. Grip strength was measured by dynamometer maximal strength and rapid force processing. No arm flexion or swinging, squatting, bending or foot-stomping were allowed when measuring force. For proper use, care had to be taken to ensure that the grip dynamometer did not touch the body or clothing during the evaluation, and the highest value was recorded by taking 2 measurements at least 15 s apart. Depression of patients was assessed using the Geriatric Depression Scale-15 (GDS-15), a simplified version of the 15-question GDS [16]. In this case, the patients answered questions by yes or no, obtaining a score of 1 or 0, respectively, with a maximum total score of 15. The higher the score was, the greater the degree of depression would be implicated. A score equal to or greater than 5 indicated depression, while a score of 0–4 suggested no illness. Social support was measured with the SSRS scale, which involved three objective supports (4–16 points), four subjective supports (5–38 points), and three levels of social support (3–12 points). The higher the total score was, the higher the level of social support became. The reliability and validity of the SSRS scale were considered satisfactory [17]. According to the results, social support was rated as low or high. Comorbidities were measured using the Charlson Comorbidity Index (CCI), which was adapted to assess common diseases in older adults and weighted as per its potential impact on outcomes [18].

### Statistical methods

Statistical analysis and mapping were performed using SPSS 22.0 (IBM, Armonk, New York, USA) and R 4.1.1 for Windows (Lucent Technologies, Jasmine Hill, New Jersey, USA). Measurement data with normal distribution were presented as mean ± standard deviation (SD), while non-normally distributed data were expressed as quartiles. Independent-sample t-test was applied to compare age, BMI, CCI, Grip strength, calf circumference, serum albumin and other blood and biochemical routine parameters between the two groups. Chi-square test was used to compare the count data. The risk factors for cognitive frailty in elderly patients with CKD were analyzed. For this purpose, a univariate analysis was performed to statistically derive related variables. Cognitive frailty was designed as the dependent variable and independent variables included the relevant indexes, age, sex, educational level, marital status, depression, SSRS, smoking, drinking, CCI, BMI, grip strength, calf circumference, serum albumin, total cholesterol (CHOL), low density lipoprotein (LDL), eGFR, hemoglobin (HGB), and albuminuria. Multivariate logistic regression analysis was performed to identify independent risk factors for cognitive frailty in older adults with CKD and useful factor combinations that could be used to predict cognitive frailty. The variables that were found statistically significant in univariate analysis were employed as independent variables in multivariate logistic regression analysis (α_in_ = 0.05, α_out_ = 0.10). *P*-values < 0.05 were considered statistically significant. All probabilities were two-tailed. The relevant influencing factors were further employed to establish a nomogram for predicting the risk of cognitive frailty in older patients with CKD. Receiver operating characteristic (ROC) curve was drawn to judge the discriminative capability of the prediction model. An area under the curve (AUC) of greater than 0.75 indicated that the prediction model had a good discrimination ability. In logistic regression predictive models, the concordance index (C-index), defined as the AUC of ROC, which was a numerical measure of discriminative power used for reflecting predictive accuracy. Then, calibration plots curve with bootstrap samples were drawn, which was a graphical evaluation of predictive power to compare the observed probabilities with those predicted by the nomogram.

## Results

### General characteristics of patients

**Fig. 1:**
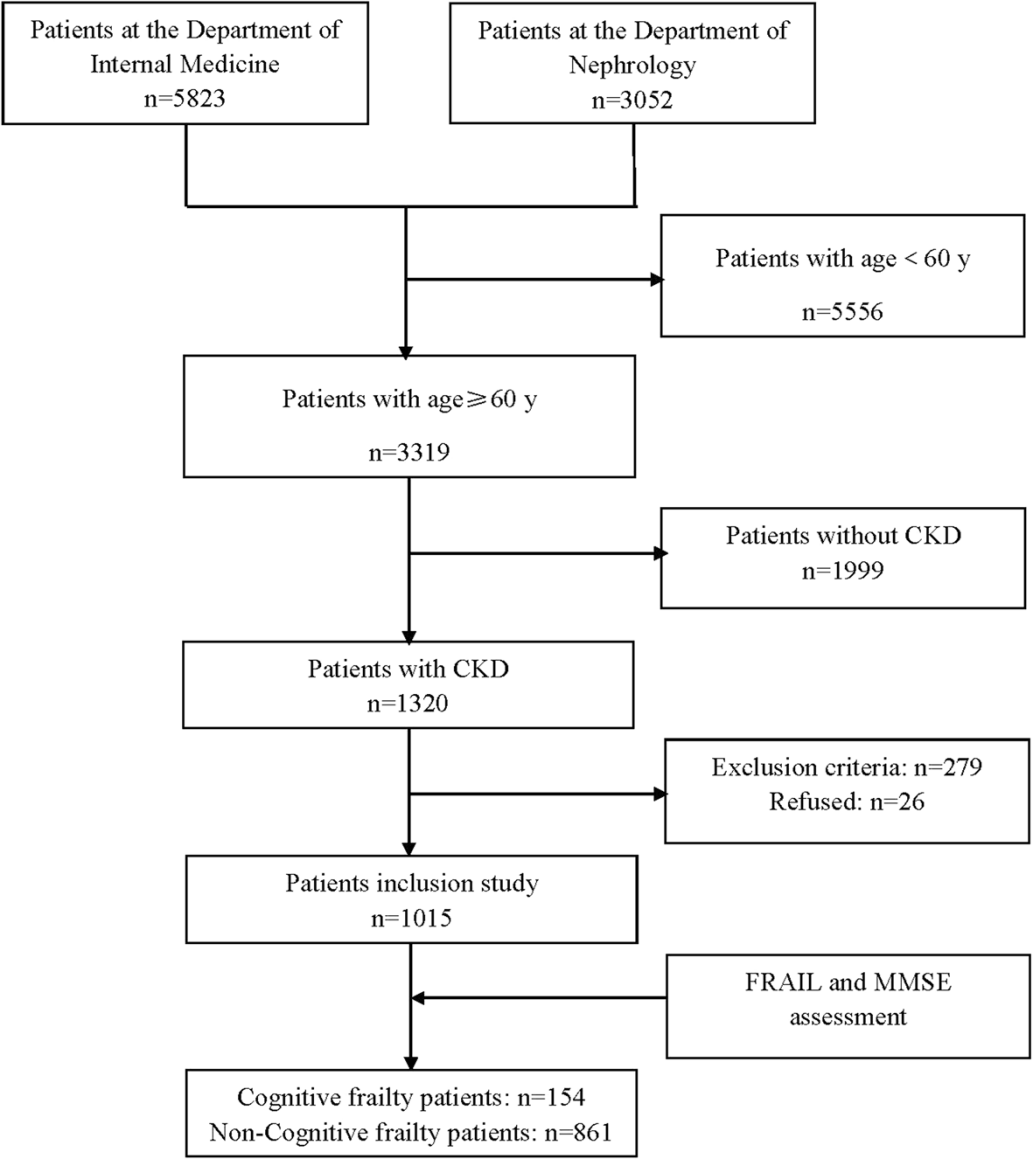
A flow chart of screening

**Fig. 2:**
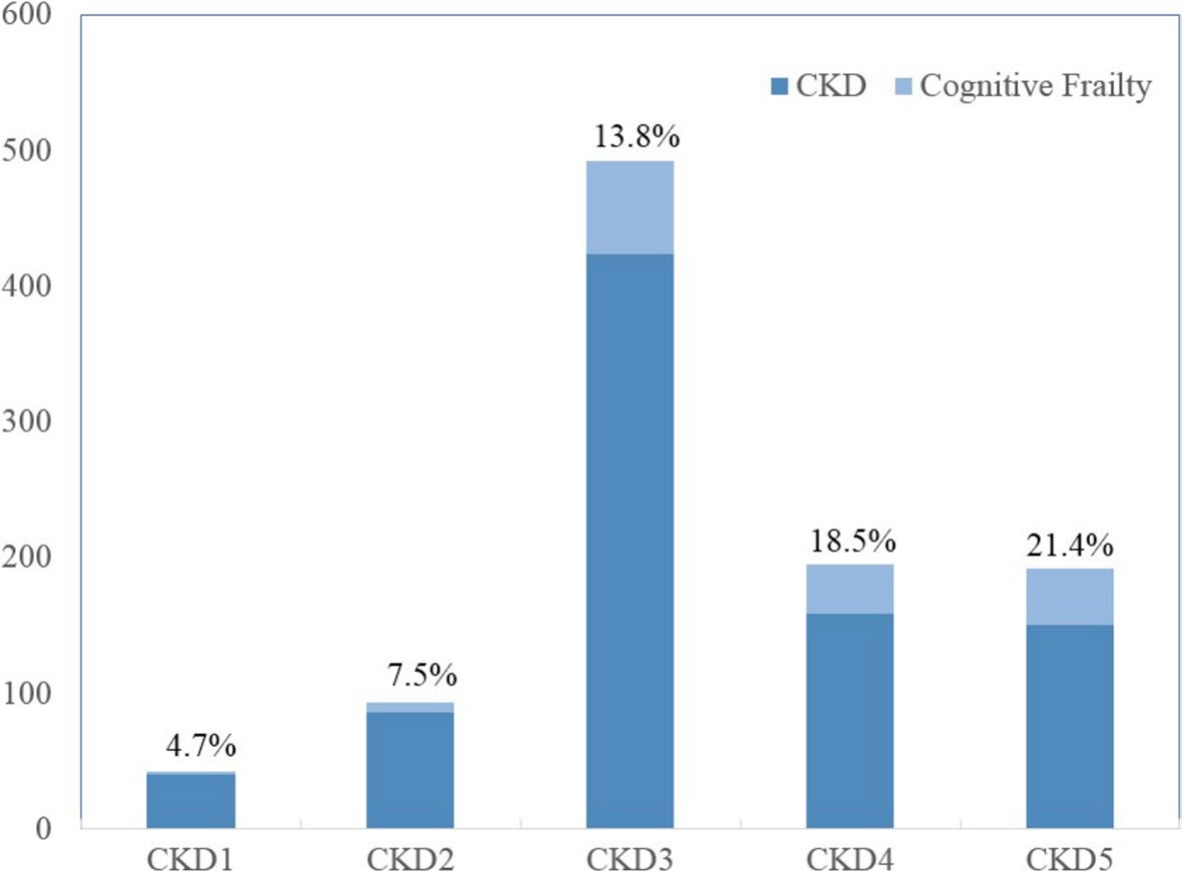
Prevalence of cognitive frailty in CKD patients at stages 1–5

As shown in the screening flow chart (Fig. 1), a total of 1,015 elderly CKD patients out of 8875 inpatients were enrolled, with an age ranging from 60 to 98 years (mean 77.3 ± 9.2 years). Based on FRAIL and MMSE assessments, 154 (15.2%) patients were categorized in the cognitive frailty group and 861 (84.8%) patients were in the non-cognitive frailty group. Among those with cognitive frailty, 87 patients (56.5%) were males and 67 (43.5%) were females. In the non-cognitive frailty group, 520 (60.4%) were male and 341 (39.6%) were female. As shown in Fig. 2, the number of CKD cases with cognitive frailty at stages 1–5 was 2, 7, 68, 36, and 41, respectively, with a prevalence of 4.7%, 7.5%, 13.8%, 18.5%, and 21.4%, respectively. Compared with the non-cognitive frailty group, those with cognitive frailty featured older age (82.0 ± 8.6 *vs* 76.5 ± 9.0, *p* < 0.01), a lower frequency of having spouses (60.4% *vs* 80.7%, *p* < 0.01), lower BMI (23.23 ± 3.67 vs 25.07 ± 3.80, *p* < 0.01), higher frequency of depression (45.5% vs 14.1%, *p* < 0.01), higher frequency of albuminuria (33.8% vs 17.9%, *p* < 0.01), higher CCI (6.59 ± 1.93 vs 3.11 ± 1.72, *p* < 0.01), lower grip strength (14.73 ± 5.84 vs 21.28 ± 7.01, *p* < 0.01), and lower calf circumference (31.64 ± 5.73 vs 34.95 ± 5.23, *p* < 0.01), as shown in Table 1.

**Table 1:** Characteristics of cognitive frailty in patients with chronic kidney disease

	Cognitive frailty (*n* = 154)	Non-Cognitive frailty (*n* = 861)	*P* value
Age (years)	82.0 ± 8.6	76.5 ± 9.0	** < 0.001**
Sex, n(%)			
Male	87 (56.5%)	520 (60.4%)	0.363
Female	67 (43.5%)	341(39.6%)	
Marital status, n(%)			
Married	93(60.4%)	695(80.7%)	** < 0.001**
Divorced /Widowed	61(39.6%)	166(19.3%)	
BMI (kg/m^2^)	23.23 ± 3.67	25.07 ± 3.80	** < 0.001**
Smoking	53 (34.4%)	308 (35.8%)	0.746
Drinking, n(%)	40 (26.0%)	198(23.0%)	0.422
Educational level			
illiteracy, n(%)	16(10.4%)	17(2.0%)	** < 0.001**
Primary school, n(%)	36(23.4%)	133(15.4%)	
Junior high school, n(%)	48(31.2%)	350(40.7%)	
High school or above, n(%)	54(35.4%)	361(41.9%)	
SSRS, n(%)			
Low level, n(%)	71(46.1%)	152(17.8%)	** < 0.001**
High level, n(%)	83(53.9%)	708(82.2%)	
Depression, n(%)	70(45.5%)	121(14.1%)	** < 0.001**
CCI	6.59 ± 1.93	3.11 ± 1.72	** < 0.001**
Grip strength(kg)	14.73 ± 5.84	21.28 ± 7.01	** < 0.001**
Calf circumference(cm)	31.64 ± 5.73	34.95 ± 5.23	** < 0.001**
Serum albumin (g/L)	35.22 ± 5.27	36.16 ± 5.67	**0.056**
CHOL(mmol/L)	4.14 ± 1.17	4.45 ± 1.41	**0.010**
LDL(mmol/L)	1.85 ± 0.84	2.06 ± 1.26	0.053
eGFR(ml/min)	31.88 ± 20.94	39.87 ± 24.20	** < 0.001**
HGB(g/L)	106.78 ± 20.93	114.09 ± 21.01	** < 0.001**
Albuminuria, n(%)	52 (33.8%)	154 (17.9%)	** < 0.001**

*BMI* Body mass index, *SSRS* Social Support Rating Scale, *CCI* Charlson Comorbidity Index, *CHOL* total Cholesterol, *HDL* High density lipoprotein, *LDL* Low density lipoprotein, *eGFR* estimated Glomerular filtration rate, *HGB* Hemoglobin

### Risk factors for cognitive frailty in elderly patients with CKD

Univariate logistic regression analysis was conducted to identify the risk factors for cognitive frailty (Table 2). Age, education, marital status, depression, social support, comorbidity index, BMI, grip strength, calf circumference, total cholesterol, eGFR, HGB and albuminuria were found statistically significant variables in univariate analysis. Then, multivariate logistic regression analysis was performed to identify the independent risk factors for cognitive frailty, and a stepwise method was adopted to identify the useful combination of factors that could predict cognitive frailty (Table 3). As a result, age (OR = 1.11, 95%CI 1.08–1.14, *p* < 0.001), depression (OR = 2.52, 95%CI 1.54–4.11, *p* < 0.001), low social support (OR = 2.08, 95%CI 1.28–3.39, *p* = 0.003), CCI (OR = 1.92, 95%CI 1.70–2.18, *p* < 0.001), eGFR (OR = 0.98, 95%CI 0.96–0.99, *p* < 0.001) and albuminuria (OR = 5.93, 95%CI 3.28–10.74, *p* < 0.001) were identified as independent risk factors for cognitive frailty in older CKD patients.

**Table 2:** Univariate Logistic regression analysis of cognitive frailty in older patients with CKD

Variables	*β*	*SE*	*WaldX*^*2*^	*P*	*OR*	95%*CI*
Age	0.07	0.01	45.16	< 0.001	1.08	1.05–1.10
Sex	0.16	0.18	0.83	0.363	1.17	0.83–1.66
Educational level	-0.44	0.10	18.83	< 0.001	0.64	0.53–0.79
Marital status	-1.01	0.19	29.49	< 0.001	0.36	0.25–0.52
Depression	1.63	0.19	74.07	< 0.001	5.10	3.52–7.39
SSRS	1.38	0.19	55.54	< 0.001	3.96	2.76–5.68
Smoking	-0.06	0.18	0.11	0.746	0.94	0.66–1.35
Drinking	0.16	0.20	0.64	0.422	1.18	0.79–1.74
CCI	0.64	0.05	152.68	< 0.001	1.89	1.71–2.09
BMI	-0.14	0.03	29.32	< 0.001	0.87	0.83–0.92
Grip strength	-0.14	0.02	72.50	< 0.001	0.87	0.85–0.90
Calf circumference	-0.09	0.02	29.15	< 0.001	0.92	0.89–0.95
Serum albumin	-0.03	0.02	3.63	0.057	0.972	0.944–1.001
CHOL	-0.20	0.08	6.70	0.010	0.822	0.71–0.95
LDL	-0.16	0.08	3.74	0.053	0.85	0.72–1.00
eGFR	-0.02	0.01	14.39	< 0.001	0.99	0.98–0.99
HGB	-0.02	0.01	15.29	< 0.001	0.98	0.98–0.99
albuminuria	0.85	0.19	19.57	< 0.001	2.34	1.61–3.41

*BMI* Body mass index, *SSRS* Social Support Rating Scale (low), *CCI* Charlson Comorbidity Index, *CHOL* total Cholesterol, *HDL* High density lipoprotein, *LDL* Low density lipoprotein, *eGFR* estimated Glomerular filtration rate, *HGB* Hemoglobin

**Table 3:** Multivariate Logistic regression analysis of risk factors associated with cognitive frailty in older patients with CKD

Variables	*Β*	*SE*	*WaldX*^*2*^	*P*	*OR*	95%*CI*
Age	0.10	0.02	46.44	0.001	1.11	1.08–1.14
Depression	0.92	0.25	13.62	0.001	2.52	1.54–4.11
SSRS	0.73	0.25	8.75	0.003	2.08	1.28–3.39
CCI	0.66	0.06	104.85	< 0.001	1.92	1.70–2.18
eGFR	-0.02	0.01	15.69	< 0.001	0.98	0.96–0.99
albuminuria	1.78	0.30	34.56	< 0.001	5.93	3.28–10.74

*SSRS* Social Support Rating Scale (low), *CCI* Charlson Comorbidity Index, *eGFR* estimated Glomerular filtration rate, *HGB* Hemoglobin

**Fig. 3:**
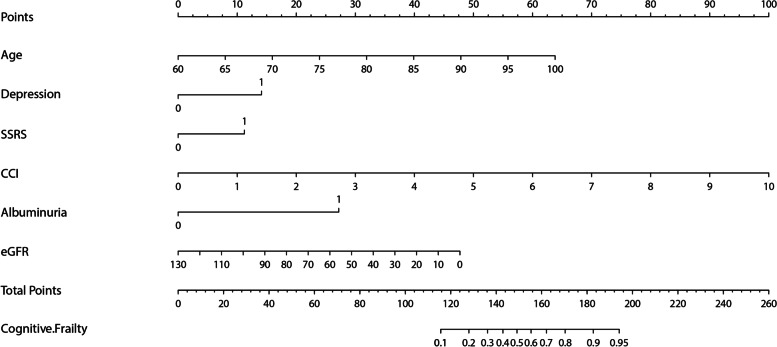
The nomogram for predicting cognitive frailty in elderly patients with CKD, based on a multivariate regression model. CCI-Charlson comorbidity index, eGFR: estimated Glomerular filtration rate; SSRS- Social Support Rating Scale (low)

**Fig. 4:**
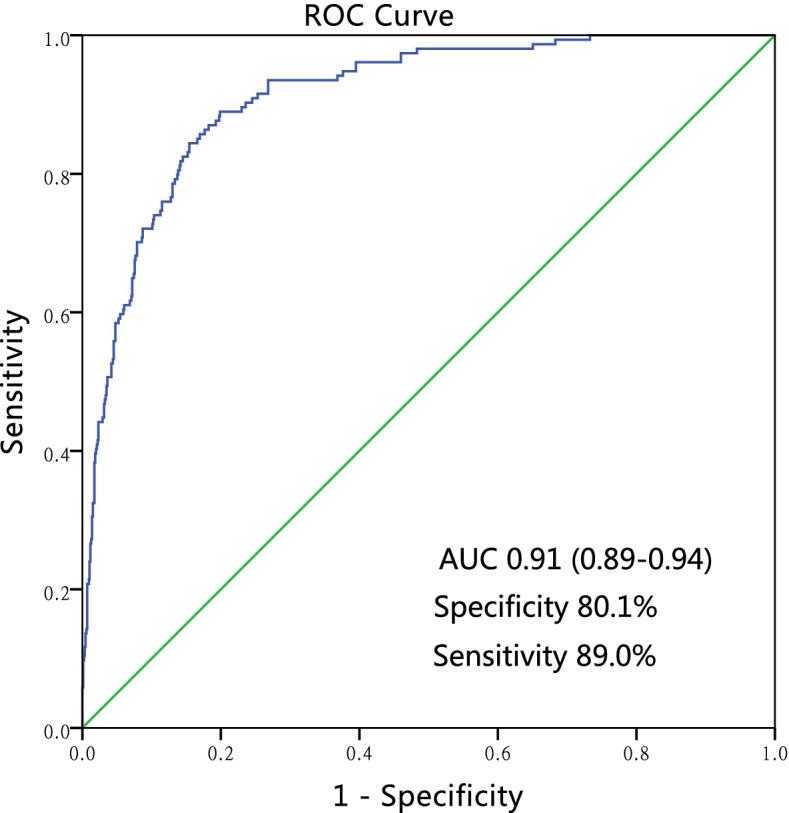
Receiver operating characteristic (ROC) curve for the prediction model. AUC: area under the curve

**Fig. 5:**
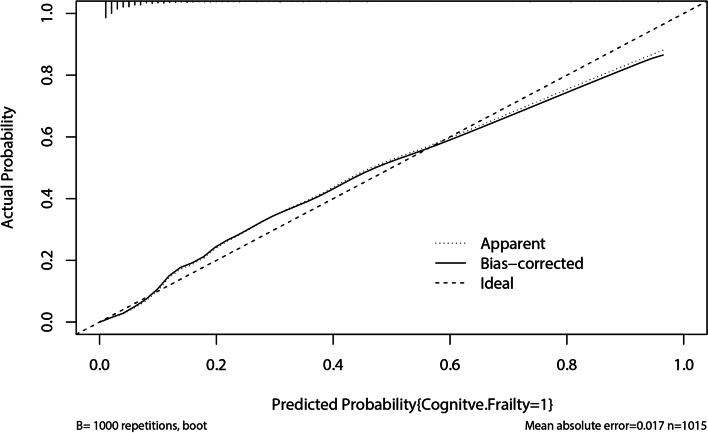
Calibration curve for the cognitive frailty prediction model in elderly CKD patients

Finally, a nomogram for predicting cognitive frailty was established based on the multivariate logistic regression model (Fig. 3). The nomogram could be applied to estimate the risk of cognitive frailty for each patient in clinical practice. ROC curve (Fig. 4) was drawn to judge the discriminative power of the cognitive frailty prediction model. The AUC was 0.91 (95%CI 0.89–0.94), indicating that the prediction model had a good discrimination ability. Calibration plots with bootstrap samples were drawn (Fig. 5). The x-axis represented the predicted probability of cognitive frailty, and the y-axis denoted the observed probability of cognitive frailty. The results indicated that the predictions made by the model were close to the observed outcomes, further demonstrating the reliability of the nomogram in estimating the risk of cognitive frailty in older CKD patients.

## Discussion

Cognitive frailty constitutes a serious threat to the quality of life and health of the elderly population. CKD is one of the most common chronic diseases, and the prevalence of frailty is higher in older adults with CKD. Frailty and cognitive impairment are closely correlated and interact in a vicious cycle, which may increase the risk of adverse outcomes such as disability, falls, hospitalization, and death. Nevertheless, multidimensional interventions incorporating physical exercise, cognitive stimulation, dietary counselling psychosocial support and microvascular health perhaps improve the prognosis of older CKD patients complicated with cognitive frailty [11, 19].

In this observational study, a total of 1,015 consecutive patients aged 60 years or above who suffered CKD and frailty were enrolled for a cognitive function assessment. Our results suggested that the prevalence of cognitive frailty in older adults with CKD was 15.2%, which was within the previously reported data range of 2.5–50% [9]. The large difference in prevalence was due to different methods of assessment used in different studies. At present, there is no consensus on the assessment of cognitive frailty. On the other hand, the severity of renal dysfunction is independently correlated with cognitive impairment [20]. Our study found that the risk of cognitive frailty in CKD patients may further increase with CKD progression.

We further investigated the risk factors associated with cognitive frailty in CKD elderly patients, and found that the main risk factors were age, albuminuria, eGFR comorbidity, depression, and low social support. The nomogram was drafted with these factors, and applied to estimate the risk of cognitive frailty for each patient in clinical practice. It is known that the prevalence of cognitive frailty increases with age [21, 22]. For older people, the functions of various tissues and organ systems of the body gradually undergo degenerative changes, resulting in a decline in the physiological functions and activities of the body. The organism reacts strongly to minor stimuli, leading to episodes of frailty. In addition, the weight and volume of brain tissue gradually decrease, cerebral white matter gradually atrophies, sensory receptors degenerate and the activities of synaptic connections and neurotransmitters decrease. Furthermore, cerebral vascular sclerosis, ischemia and hypoxia may arise, and the overall function of the nervous system declines, which means more frequent nerve cell degeneration, apoptosis or necrosis. All these changes predispose cognitive impairment. It was reported that lower eGFR and albuminuria were associated with cognitive decline [23], and albuminuria predicted worse memory function [24].

Another risk factor for cognitive frailty was comorbidity, which was assessed with the Charlson Comorbidity Index. It was suggested that the more severe comorbidity was, the more likely cognitive frailty would occur. Patients with CKD often suffer from multiple chronic diseases, which together increase the risk of frailty and cognitive frailty. This process involves physiological decline and a decrease in the reserve function of multiple systems throughout the body, affecting the daily activities of the patients [25]. In addition, CKD patients are more susceptible to vasculopathy, vitamin D deficiency, and protein-energy depletion. As a result, muscle mass and strength are affected, along with abnormal neurotrophic factor metabolism, leading to cerebral white matter damage, reduced white matter integrity, and accumulation of neurotoxic amyloid in the brain. Due to this physiological scenario, patients are at increased risk to develop physical and cognitive frailty. Therefore, attention should be paid to the management of comorbidities in clinical practice, including timely assessment and intervention, in order to control or reverse the risk factors for cognitive frailty and further delay the development of this cognitive impairment.

This study found that depression and reduced social support were independent risk factors for cognitive frailty. In particular, depressed patients were 2.5 times more likely to develop this condition than non-depressed patients. In turn, those with poor access to social support were about 2.1 times more likely to develop cognitive frailty than patients with better social support. In this regard, Rivan et al. analyzed a population of Malaysian seniors in a 5-year follow-up study [26] and discovered that the prevalence of cognitive frailty was 7.1%, with depression being one of the major risk factors (OR = 1.20, 95%CI: 1.05–1.37, *p* < 0.05). Depression has been shown to be strongly associated with cognitive dysfunction and frailty, which may be due to the common risk factors and similar pathological basis, including oxidative stress, chronic inflammation, cerebral white matter lesions, and mitochondrial dysfunction [19, 27].

Poor social support may produce psychological and emotional consequences. Lack of moral support and social engagement can lead to weak volition and retardation of thinking in patients. Eventually, their social interaction decreases and potentially develop cognitive frailty. Quality social support can reduce the occurrence of depression and cognitive frailty [28]. Therefore, health care professionals should fully assess the psychological status of their patients and consider the degree to which they feel social support. Providing targeted psychological therapy and encouraging patients to actively participate in social activities are important to further prevent the development of cognitive frailty.

The strength of this study was the nomogram based on the above risk factors. The nomogram prediction model has the advantage of visualization. It can predict the cognitive frailty quickly and accurately based on clinical data. The probability of cognitive frailty in older patients with CKD can be obtained easily by using a series of convenient clinical data.

This study was limited by a cross-sectional single-center design and a relatively small sample size, The prediction model established in this study needed to be further validated. The study samples were collected in one region, further investigations at different regions were warranted.

## Conclusions

The prevalence of cognitive frailty was high in older patients with CKD. Advanced age, comorbidity, depression, reduced social support, eGFR and albuminuria were independent risk factors for this condition. For earlier diagnosis and better clinical prognosis, cognitive frailty assessment should be highlighted in older CKD with these characteristics.

## Data Availability

The datasets of the current study are available from the corresponding author on reasonable request.

## Abbreviations

AUC: Area under the curve
BMI: Body mass index
CCI: Charlson Comorbidity Index
CHOL: Total cholesterol
CKD: Chronic kidney disease
eGFR: Estimated Glomerular filtration rate
GDS: Geriatric Depression Scale
HDL: High density lipoprotein
HGB: Hemoglobin
IAGG: International Association of Gerontology and Geriatrics
IANA: International Academy on Nutrition and Aging
KIDIGO: Kidney Disease Improving Global Outcomes
LDL: Low density lipoprotein
MMSE: Mini-Mental State Examination,
OR: Odds ratio
SSRS: Social Support Rating Scale

## References

[1] The main data of the seventh National Census tCPsGotPsRoC, http://www.gov.cn/xinwen/2021-05/11/content_5605760.htm.

[2] Hoogendijk EO, Afilalo J, Ensrud KE, Kowal P, Onder G, Fried LP (2019). Frailty: implications for clinical practice and public health. Lancet.

[3] Cunha AIL, Veronese N, de Melo BS, Ricci NA (2019). Frailty as a predictor of adverse outcomes in hospitalized older adults: a systematic review and meta-analysis. Ageing Res Rev.

[4] Dent E, Martin FC, Bergman H, Woo J, Romero-Ortuno R, Walston JD (2019). Management of frailty: opportunities, challenges, and future directions. Lancet.

[5] Robertson DA, Savva GM, Kenny RA (2013). Frailty and cognitive impairment–a review of the evidence and causal mechanisms. Ageing Res Rev.

[6] Avila-Funes JA, Amieva H, Barberger-Gateau P, Le Goff M, Raoux N, Ritchie K (2009). Cognitive impairment improves the predictive validity of the phenotype of frailty for adverse health outcomes: the three-city study. J Am Geriatr Soc.

[7] Kelaiditi E, Cesari M, Canevelli M, van Kan GA, Ousset PJ, Gillette-Guyonnet S (2013). Cognitive frailty: rational and definition from an (I.A.N.A./I.A.G.G.) international consensus group. J Nutr Health Aging.

[8] Ruan Q, Yu Z, Chen M, Bao Z, Li J, He W (2015). Cognitive frailty, a novel target for the prevention of elderly dependency. Ageing Res Rev.

[9] Bu Z, Huang A, Xue M, Li Q, Bai Y, Xu G (2021). Cognitive frailty as a predictor of adverse outcomes among older adults: a systematic review and meta-analysis. Brain Behav.

[10] Zhang L, Wang F, Wang L, Wang W, Liu B, Liu J (2012). Prevalence of chronic kidney disease in China: a cross-sectional survey. Lancet.

[11] Shen Z, Ruan Q, Yu Z, Sun Z (2017). Chronic kidney disease-related physical frailty and cognitive impairment: a systemic review. Geriatr Gerontol Int.

[12] Stevens PE, Levin A (2013). Evaluation and management of chronic kidney disease: synopsis of the kidney disease: improving global outcomes 2012 clinical practice guideline. Ann Intern Med.

[13] Morley JE, Malmstrom TK, Miller DK (2012). A simple frailty questionnaire (FRAIL) predicts outcomes in middle aged African Americans. J Nutr Health Aging.

[14] Folstein MF, Folstein SE, McHugh PR (1975). "Mini-mental state". A practical method for grading the cognitive state of patients for the clinician. J Psychiatr Res.

[15] Levey AS, Stevens LA, Schmid CH (2009). A new equation to estimate glomerular filtration rate. Ann Intern Med.

[16] Cruice M, Worrall L, Hickson L (2011). Reporting on psychological well-being of older adults with chronic aphasia in the context of unaffected peers. Disabil Rehabil.

[17] Xiao SY (1994). Theory and application of social support scale. J Clin Psychol Med.

[18] Bannay A, Chaignot C, Blotière PO, Basson M, Weill A, Ricordeau P (2016). The best use of the charlson comorbidity index with electronic health care database to predict mortality. Med Care.

[19] Asam M, Indranil D (2021). Chronic Kidney disease and cognitive impairment. J Stroke Cerebrovasc Dis.

[20] Coppolino G, Bolignano D, Gareri P, Ruberto C, Andreucci M, Ruotolo G (2018). Kidney function and cognitive decline in frail elderly: two faces of the same coin?. Int Urol Nephrol.

[21] Fabrício DM, Chagas MHN, Diniz BS (2020). Frailty and cognitive decline. Transl Res.

[22] Sugimoto T, Arai H, Sakurai T (2022). An update on cognitive frailty: Its definition, impact, associated factors and underlying mechanisms, and interventions. Geriatr Gerontol Int.

[23] Drew DA, Weiner DE, Sarnak MJ (2019). Cognitive impairment in CKD: pathophysiology, management, and prevention. Am J Kidney Dis.

[24] Sacre JW, Magliano DJ, Zimmet PZ, Polkinghorne KR, Chadban SJ, Anstey KJ (2019). Associations of chronic kidney disease markers with cognitive function: a 12-year follow-up study. J Alzheimers Dis.

[25] Walker SR, Wagner M, Tangri N (2014). Chronic kidney disease, frailty, and unsuccessful aging: a review. J Ren Nutr.

[26] Rivan NFM, Shahar S, Rajab NF, Singh DKA, Che Din N, Mahadzir H (2020). Incidence and predictors of cognitive frailty among older adults: a community-based longitudinal study. Int J Environ Res Public Health.

[27] Jung S, Lee YK, Choi SR, Hwang SH, Noh JW (2013). Relationship between cognitive impairment and depression in dialysis patients. Yonsei Med J.

[28] Duppen D, Van der Elst MCJ, Dury S, Lambotte D, De Donder L (2019). The social environment's relationship with frailty: evidence from existing studies. J Appl Gerontol.

